# Complete genome sequence of *Ferroglobus placidus* AEDII12DO

**DOI:** 10.4056/sigs.2225018

**Published:** 2011-10-01

**Authors:** Iain Anderson, Carla Risso, Dawn Holmes, Susan Lucas, Alex Copeland, Alla Lapidus, Jan-Fang Cheng, David Bruce, Lynne Goodwin, Samuel Pitluck, Elizabeth Saunders, Thomas Brettin, John C. Detter, Cliff Han, Roxanne Tapia, Frank Larimer, Miriam Land, Loren Hauser, Tanja Woyke, Derek Lovley, Nikos Kyrpides, Natalia Ivanova

**Affiliations:** 1 DOE Joint Genome Institute, Walnut Creek, California, USA; 2 Microbiology Department, University of Massachusetts, Amherst, Massachusetts, USA; 3 Los Alamos National Laboratory, Los Alamos, New Mexico, USA; 4 Biosciences Division, Oak Ridge National Laboratory, Oak Ridge, Tennessee, USA

**Keywords:** *Archaea*, *Euryarchaeota*, *Archaeoglobales*, hydrothermal vent, hyperthermophile, anaerobe

## Abstract

*Ferroglobus placidus* belongs to the order *Archaeoglobales* within the archaeal phylum *Euryarchaeota*. Strain AEDII12DO is the type strain of the species and was isolated from a shallow marine hydrothermal system at Vulcano, Italy. It is a hyperthermophilic, anaerobic chemolithoautotroph, but it can also use a variety of aromatic compounds as electron donors. Here we describe the features of this organism together with the complete genome sequence and annotation. The 2,196,266 bp genome with its 2,567 protein-coding and 55 RNA genes was sequenced as part of a DOE Joint Genome Institute Laboratory Sequencing Program (LSP) project.

## Introduction

Strain AEDII12DO (=DSM 10642) is the type strain of the species *Ferroglobus placidus*. It was isolated from a shallow hydrothermal vent system at Vulcano Island, Italy [1]. *F. placidus* is metabolically quite versatile. It was isolated based on its ability to use ferrous iron as an electron donor, and was also shown to use hydrogen and sulfide as electron donors, with nitrate or thiosulfate as electron acceptors [1]. Subsequently, it was shown to produce N_2_O from nitrite, which is an unusual ability for an anaerobic organism [2]. It can also oxidize acetate and several aromatic compounds using ferric iron as the electron acceptor [3,4]. *F. placidus* is the first archaeon found to anaerobically oxidize aromatic compounds [4]. The genes and pathways involved in degradation of benzene, benzoate, and phenol have been recently characterized [5,6].

**Figure 1:**
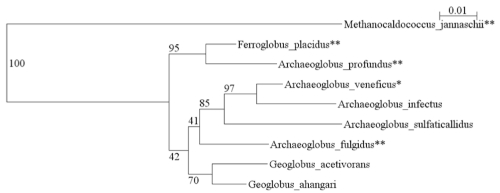
16S ribosomal RNA phylogenetic tree of *Archaeoglobaceae*. The tree was generated with weighbor [9] through the Ribosomal Database Project website [10] and displayed with njplot [11]. Organisms with two asterisks after the name are those with complete genomes sequenced and published. Those with one asterisk have a genome project in progress, according to the Genomes OnLine Database [12]. *Methanocaldococcus jannaschii* is the outgroup.

*F. placidus* is the only species in the genus *Ferroglobus*. It belongs to the family *Archaeoglobaceae*, which also contains the genera *Archaeoglobus* and *Geoglobus*. Genome sequences have been published for *A. fulgidus* and *A. profundus* [7,8]. Figure 1 shows the phylogenetic relationships between members of the family *Archaeoglobaceae*.

## Organism information

*F. placidus* was isolated from a mixture of sand and water at a beach close to Vulcano Island, Italy [1]. The sample was taken from a depth of 1 m; the temperature of the sample was 95°C and the pH was 7.0 [1]. A 1.0 mL aliquot of the sample was incubated in FM medium at 85°C with shaking. The medium contained FeS as an electron donor [1]. *F. placidus* was isolated from the enrichment culture using optical tweezers [1]. The cells are irregular cocci with a triangular shape, and one or two flagella were present [1]. Growth occurred between 65°C and 95°C with an optimum of 85°C [1]. The optimal pH for growth was 7.0, and growth was observed over a range of 6.0 ­to 8.5 [1]. The optimal salinity for growth was 2.0%, with growth occurring between 0.5 and 4.5% NaCl [1]. *F. placidus* could use ferrous iron, hydrogen, or sulfide as electron donors and nitrate or thiosulfate as electron acceptors [1]. *F. placidus* also can anaerobically oxidize aromatic compounds with ferric iron as electron acceptor. The aromatic compounds it can utilize include benzene, benzoate, phenol, 4-hydroxybenzoate, benzaldehyde, p-hydroxybenzaldehyde and t-cinnamic acid [4,5]. The features of the organism are listed in Table 1.

**Table 1:** Classification and general features of *F. placidus* AEDII12DO according to the MIGS recommendations [13].

MIGS ID	Property	Term	Evidence code^a^
	Current classification	Domain *Archaea*	TAS [14]
		Phylum *Euryarchaeota*	TAS [15]
		Class *Archaeoglobi*	TAS [16,17]
		Order *Archaeoglobales*	TAS [17,18]
		Family *Archaeoglobaceae*	TAS [17,19]
		Genus *Ferroglobus*	TAS [1,20]
		Species *Ferroglobus placidus*	TAS [1,20]
		Type strain AEDII12DO	TAS [1]
	Cell shape	irregular coccus	TAS [1]
	Motility	motile	TAS [1]
	Sporulation	nonsporulating	NAS
	Temperature range	65-95°C	TAS [1]
	Optimum temperature	85°C	TAS [1]
MIGS-6.3	Salinity	0.5-4.5% NaCl (optimum 2%)	TAS [1]
MIGS-22	Oxygen requirement	anaerobe	TAS [1]
	Carbon source	CO_2_	TAS [1]
	Energy metabolism	chemolithotrophic, chemoorganotrophic	TAS [1,3,4]
MIGS-6	Habitat	marine geothermally heated areas	TAS [1]
MIGS-15	Biotopic relationship	free-living	TAS [1]
MIGS-14	Pathogenicity	none	NAS
	Biosafety level	1	NAS
	Isolation	geothermally heated sediment	TAS [1]
MIGS-4	Geographic location	Vulcano island, Italy	TAS [1]
MIGS-5	Isolation time	unknown	
MIGS-4.1 MIGS-4.2	Latitude Longitude	38.4154 14.9609	TAS [1]
MIGS-4.3	Depth	1 m	TAS [1]
MIGS-4.4	Altitude	not applicable	

a) Evidence codes – IDA: Inferred from Direct Assay; TAS: Traceable Author Statement (i.e., a direct report exists in the literature); NAS: Non-traceable Author Statement (i.e., not directly observed for the living, isolated sample, but based on a generally accepted property for the species, or anecdotal evidence). These evidence codes are from the Gene Ontology project [21].

## Genome sequencing information

### Genome project history

This organism was selected for sequencing based on its phylogenetic position and its phenotypic differences from other members of the family *Archaeoglobaceae*. It is part of a Laboratory Sequencing Program (LSP) project to sequence diverse archaea. The genome project is listed in the Genomes On Line Database [12] and the complete genome sequence has been deposited in GenBank. Sequencing, finishing, and annotation were performed by the DOE Joint Genome Institute (JGI). A summary of the project information is shown in Table 2.

**Table 2:** Genome sequencing project information

**MIGS ID**	**Property**	**Term**
MIGS-31	Finishing quality	Finished
MIGS-28	Libraries used	Illumina standard library, 454 standard library, 454 15 kb paired end library
MIGS-29	Sequencing platforms	Illumina GA II, 454 GS FLX Titanium
MIGS-31.2	Sequencing coverage	Illumina 245×, 454 47×
MIGS-30	Assemblers	Velvet, Newbler, phrap
MIGS-32	Gene calling method	Prodigal, GenePRIMP
	INSDC ID	CP001899
	Genbank Date of Release	February 16, 2010
	GOLD ID	Gc01209
	NCBI project ID	33635
MIGS-13	Source material identifier	DSM 10642
	Project relevance	Phylogenetic diversity, biotechnology

### Growth conditions and DNA isolation

The strain *Ferroglobus placidus* AEDII12DO (containing plasmid XY) has been deposited in the Deutsche Sammlung von Mikroorganismen und Zellkulturen (DSMZ) by Prof. Dr. K. O. Stetter, Lehrstuhl für Mikrobiologie, Universität Regensburg, Universitätsstr. 31, D-93053 Regensburg, Germany as DSM 10642.

*F. placidus* strain AEDII12DO was obtained from the DSMZ. Strict anaerobic culturing and sampling techniques were used throughout [22,23]. Ten 100 ml bottles of *F. placidus* cells were grown with acetate (10 mM) as the electron donor, and Fe(III) citrate (56 mM) as the electron acceptor. *F. placidus* medium was prepared as previously described [4]. After autoclaving, FeCl_2_ (1.3 mM), Na_2_SeO_4_ (30 µg/L), Na_2_WO_4_ (40 µg/L), APM salts (1 g/L MgCl_2_, 0.23 g/L CaCl_2_**),** DL vitamins [24] and all electron donors were added to the sterilized medium from anaerobic stock solutions. Cultures were incubated under N_2_:CO_2_ (80:20) at 85 °C in the dark.

For extraction of DNA, cultures (100 ml in 156 ml serum bottles) were divided into 50 ml conical tubes (Falcon), and cells were pelleted by centrifugation at 3,000 x g for 20 minutes. Cell pellets were resuspended in 10 ml TE sucrose buffer (10 mM Tris, pH 8.0, 1 mM EDTA, and 6.7% sucrose). The resuspended cells were distributed into 10 different 2 ml screw cap tubes and 3 µl Proteinase K (20 mg/ml), 30 µl sodium dodecyl sulfate (10% solution), and 10 µl RNase A (5 ug/ul) were added to each tube. Tubes were incubated at 37ºC for 20 min, and centrifuged at 16,100 x g for 15 minutes. The supernatant was transferred to a new set of tubes and 600 µl phenol (TE saturated, pH 7.3), and 400 μl chloroform-isoamyl alcohol were added. These tubes were then mixed on a Labquake rotator (Barnstead/Thermolyne, Dubuque, Iowa) for 10 min and centrifuged at 16,100 x g for 5 min. The aqueous layer was removed and transferred to new 2-ml screw cap tubes. The phenol/chloroform extraction step was performed again. The aqueous layer was transferred to a new tube, and 100 µl 5 M ammonium acetate, 20 µl glycogen (5 mg/ml; Ambion), and 1 ml cold (-20 °C) isopropanol (Sigma) were added. Nucleic acids were precipitated at -30 °C for 1 hour and pelleted by centrifugation at 16,100 x g for 30 min. The pellet was then cleaned with cold (-20 °C) 70% ethanol, dried, and resuspended in sterile nuclease-free water (Ambion).

### Genome sequencing and assembly

The genome of *F. placidus* was sequenced at the Joint Genome Institute using a combination of Illumina and 454 technologies. An Illumina GAII shotgun library with reads of 539 Mb, a 454 Titanium draft library with average read length of 292.3 bases, and a paired-end 454 library with an average insert size of 15.5 Kb were generated for this genome. All general aspects of library construction and sequencing performed at the JGI can be found at the DOE JGI website [25].

Illumina sequencing data was assembled with Velvet [26], and the consensus sequences were shredded into 1.5 kb overlapped fake reads and assembled together with the 454 data. Draft assemblies were based on 104 Mb 454 draft data and 454 paired end data. The initial Newbler assembly contained 33 contigs in 1 scaffold. We converted the initial 454 assembly into a phrap assembly by making fake reads from the consensus, collecting the read pairs in the 454 paired end library. The Phred/Phrap/Consed software package [27] was used for sequence assembly and quality assessment [28-30] in the following finishing process. After the shotgun stage, reads were assembled with parallel phrap (High Performance Software, LLC). Possible mis-assemblies were corrected with gapResolution (Cliff Han, unpublished), Dupfinisher [31], or sequencing cloned bridging PCR fragments with subcloning or transposon bombing (Epicentre Biotechnologies, Madison, WI). Gaps between contigs were closed by editing in Consed, by PCR and by Bubble PCR primer walks. A total of 43 additional reactions were necessary to close gaps and to raise the quality of the finished sequence.

### Genome annotation

Genes were identified using Prodigal [32], followed by a round of manual curation using GenePRIMP [33]. The predicted CDSs were translated and used to search the National Center for Biotechnology Information (NCBI) nonredundant database, UniProt, TIGRFam, Pfam, PRIAM, KEGG, COG, and InterPro databases. The tRNAScanSE tool [34] was used to find tRNA genes, whereas ribosomal RNAs were found by using BLASTn against the ribosomal RNA databases. The RNA components of the protein secretion complex and the RNase P were identified by searching the genome for the corresponding Rfam profiles using INFERNAL [35]. Additional gene prediction analysis and manual functional annotation was performed within the Integrated Microbial Genomes (IMG) platform [36] developed by the Joint Genome Institute, Walnut Creek, CA, USA [37].

## Genome properties

**Figure 2:**
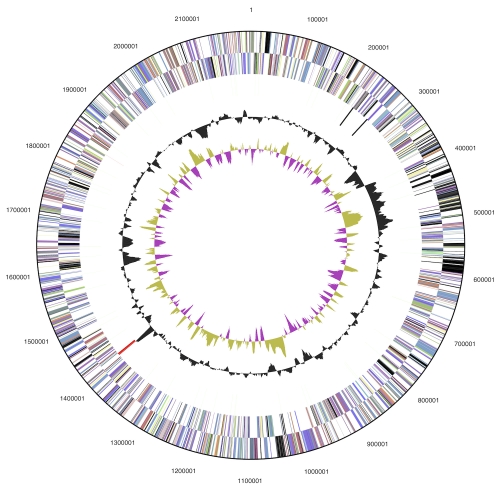
Graphical circular map of the chromosome. From outside to the center: Genes on forward strand (colored by COG categories), genes on reverse strand (colored by COG categories), RNA genes (tRNAs green, rRNAs red, other RNAs black), GC content, and GC skew.

The genome includes one circular chromosome and no plasmids, for a total size of 2,196,266 bp (Table 3 and Figure 2). This genome size is almost the same as that of *A. fulgidus* and approximately 0.6 Mbp larger than that of *A. profundus*. The mol percent G+C is 44.1%, close to the values found in the *Archaeoglobus* genomes. A total of 2,622 genes were identified, 55 RNA genes and 2,567 protein-coding genes. There are 87 pseudogenes, comprising 3.4% of the protein-coding genes. The start codon is ATG in 83.7% of the genes, GTG in 12.2%, and TTG in 5.8%. This distribution is more similar to that of *A. profundus*, to which *F. placidus* is closely related (Figure 1), than to that of *A. fulgidus*. There is one copy of each ribosomal RNA. The 5S rRNA is not found adjacent to the 16S and 23S rRNAs. Table 4 shows the distribution of genes in COG categories.

**Table 3:** Nucleotide content and gene count levels of the genome

**Attribute**	**Value**	**% of total^a^**
Size (bp)	2,196,266	100.0%
G+C content (bp)	969,331	44.1%
Coding region (bp)	1,996,425	90.9%
Number of replicons	1	
Extrachromosomal elements	0	
Total genes	2,622	
RNA genes	55	
rRNA operons	1	
Protein-coding genes	2,567	100.0%
Pseudogenes	87	3.4%
Genes with function prediction	1,619	63.1%
Genes in paralog clusters	350	13.6%
Genes assigned to COGs	1,820	70.9%
Genes assigned Pfam domains	1,889	73.6%
Genes with signal peptides	269	10.5%
Genes with transmembrane helices	502	19.6%
CRISPR repeats	6	100.0%

a) The total is based on either the size of the genome in base pairs or the total number of protein coding genes in the annotated genome

**Table 4:** Number of genes associated with the 25 general COG functional categories

**Code**	**Value**	**%age**^a^	**Description**
J	163	6.3%	Translation
A	2	0.1%	RNA processing and modification
K	90	3.5%	Transcription
L	110	4.3%	Replication, recombination and repair
B	8	0.3%	Chromatin structure and dynamics
D	23	0.9%	Cell cycle control, mitosis and meiosis
Y	0	0.0%	Nuclear structure
V	16	0.6%	Defense mechanisms
T	37	1.4%	Signal transduction mechanisms
M	42	1.6%	Cell wall/membrane biogenesis
N	24	0.9%	Cell motility
Z	0	0.0%	Cytoskeleton
W	0	0.0%	Extracellular structures
U	27	1.1%	Intracellular trafficking and secretion
O	88	3.4%	Posttranslational modification, protein turnover, chaperones
C	207	8.1%	Energy production and conversion
G	44	1.7%	Carbohydrate transport and metabolism
E	147	5.7%	Amino acid transport and metabolism
F	50	1.9%	Nucleotide transport and metabolism
H	118	4.6%	Coenzyme transport and metabolism
I	76	3.0%	Lipid transport and metabolism
P	94	3.7%	Inorganic ion transport and metabolism
Q	22	0.9%	Secondary metabolites biosynthesis, transport and catabolism
R	293	11.4%	General function prediction only
S	246	9.6%	Function unknown
-	747	29.1%	Not in COGs

a) The total is based on the total number of protein coding genes in the genome

## Insights from the genome

### Nitrogen metabolism

Some aspects of the genome of *F. placidus* have been compared with those of *A. fulgidus* and *A. profundus* [8]. Here we will focus on some additional aspects of the *F. placidus* genome. *F. placidus* has been found to use nitrate as an electron acceptor and produces N_2_O with NO as an intermediate [2]. Genes likely to encode a nitrate reductase (Ferp_0311-0314) and an adjacent nitrate transporter (Ferp_0315) were identified. Based on the experimental results, *F. placidus* is expected to have a nitric oxide-forming nitrite reductase. There are two types of this protein: cytochrome cd1 type and copper type [38]. *F. placidus* appears to lack both of these types of nitrite reductase, so it may have a new version of this enzyme. *F. placidus* was found to produce N_2_O, and it has a NorBC-type nitric oxide reductase (Ferp_1340-1341). Surprisingly it also has a nitrous oxide reductase (Ferp_0128), suggesting that under some conditions *F. placidus* may carry out complete denitrification from nitrate to N_2_.

### Central metabolism

**Figure 3:**
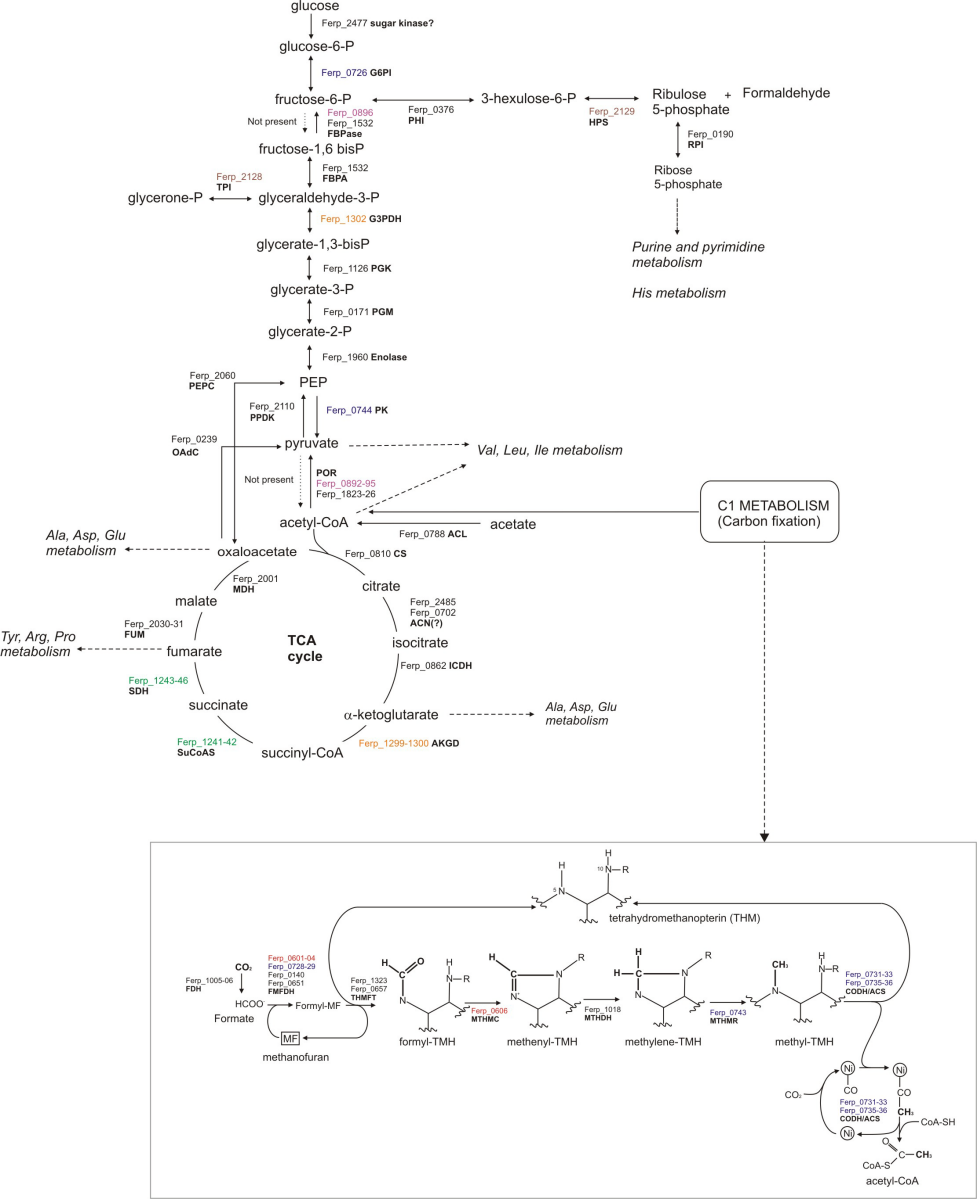
Central metabolism of the hyperthermophilic archaeon *Ferroglobus placidus*. Abbreviations: G6PI: glucose-6-phosphate isomerase. FBPase: fructose-1,6-bisphosphatase. FBPA: fructose-1,6-bisphosphate aldolase. G3PDH: glycerol-3-phosphate dehydrogenase. TPI: triose-phosphate isomerase. PGK: phosphoglycerate kinase. PGM: phosphoglycerate mutase. PEP: phosphoenolpyruvate. PEPC: phosphoenolpyruvate carboxylase. PK: pyruvate kinase. PPDK: phosphoenolpyruvate dikinase. OadC: oxaloacetate decarboxylase. POR: pyruvate ferredoxin reductase (pyruvate synthase). ACL: acetyl-CoA ligase. AP: acetate phosphatase. CS: citrate synthase. ACN: aconitase. ICDH: isocitrate dehydrogenase. AKGD: alpha-ketoglutarate dehydrogenase. SuCoAS: succinyl-CoA synthase. SDH: succinate dehydrogenase. FUM: fumarase. MDH: malate dehydrogenase. PHI: 3-hexulose-6-phosphate isomerase. HPS: 3-hexulose-6-phosphate synthase. RPI: ribose-5-phosphate isomerase. FDH: formate dehydrogenase. MF: methanofuran. FMFDH: formylmethanofuran dehydrogenase. THM: tetrahydromethanopterin. THMFT: tetrahydromethanopterin formyl transferase. MTHMC: methenyltetrahydromethanopterin cyclohydrolase. MTHMR: methylentetrahydromethanopterin reductase. CODH/ACS: CO dehydrogenase/acetyl-CoA synthase. Gene designations in colors indicates association in clusters.

*F. placidus* likely can not metabolize sugars as the Entner-Doudoroff pathway is absent from its genome, and the critical rate-limiting enzyme in the glycolysis pathway, 6-phosphofructokinase, also could not be identified. A complete gluconeogenesis pathway is present (Figure 3), including the recently discovered archaeal bifunctional fructose bisphosphate aldolase/phosphatase (Ferp_1532) [39]. A second fructose bisphosphate phosphatase may be present (Ferp_0896). Biosynthesis of C5 sugars for anabolic purposes proceeds through the reverse ribulose monophosphate pathway [40,41], in which fructose 6-phosphate is converted to hexulose 6-phosphate, from which formaldehyde is cleaved and ribulose 5-phosphate is generated.

Similar to *Archaeoglobus* species, *F. placidus* is capable of autotrophic growth. The genome contains a gene coding for the large subunit of ribulose-1,5-bisphosphate carboxylase/oxygenase (RubisCO, Ferp_1506), which fixes CO_2_ in photosynthetic organisms, but many other enzymes of the Calvin-Benson cycle are missing. *F. placidus* probably uses RubisCO as part of an AMP recycling pathway [42] rather than for carbon fixation. *F. placidus* also contains the complete acetyl-CoA reductive pathway. Based on experimental results it was predicted to use this pathway for carbon fixation [2]. This pathway is composed of a *methyl branch* that reduces CO_2_ into a methyl group by a sequence of reactions similar to those found in methanogenesis (Fig. 3, inset), and a *carbonyl branch* that converts a second CO_2_ molecule into a carbonyl group. The two moieties are then joined to form acetyl-CoA.

Interestingly, there are two full copies of pyruvate ferredoxin oxidoreductase (POR, Ferp_0892-95 and Ferp_1823-26, 32-42% identical/47-60% similar), which generates pyruvate from acetyl-CoA and CO_2_. Conversely, the genome does not contain genes coding for the pyruvate dehydrogenase complex. All of the enzymes that comprise the TCA cycle could be accounted for, with the exception of a typical aconitase. However, two genes annotated as homoaconitate hydratase (Ferp_0702 and Ferp_2485) are 40% similar to the characterized aconitase from the thermoacidophilic archaeon *Sulfolobus acidocaldarius* [43]. Also *F. placidus* has the two subunits of a predicted aconitase (Ferp_0107-0108) [44].

Even though the genes involved in central metabolism are typically scattered in the genome, it is worth noting that many of these genes are grouped in clusters in *F. placidus*. For instance, the genes coding for the formylmethanofuran dehydrogenase (FMFDH, Ferp_0601-04) are located near the methenyltetrahydromethanopterin cyclohydrolase gene (MTHMC, Ferp_0606) from the reductive acetyl-CoA pathway. Similarly, two subunits of FMFDH (Ferp_0728-29), the methylenetetrahydromethanopterin reductase gene (MTHMR, Ferp_0743), the whole CO dehydrogenase/acetyl-CoA synthase operon (CODH/ACS, Ferp_0731-33, Ferp_0735-36), and the pyruvate kinase gene (PK, Ferp_0744), are in close proximity. The operon that contains the genes coding for one of the POR complexes (Ferp_0892-96) also includes genes coding for other enzymes that belong to central metabolism, such as one of the FBPases (Ferp_0896), an ATP-NAD kinase (Ferp_0897), and shikimate dehydrogenase (Ferp_0898), which participates in the biosynthesis of aromatic amino acids.
